# Ca_V_1.3 L-type Ca^2+^ channel contributes to the heartbeat by generating a dihydropyridine-sensitive persistent Na^+^ current

**DOI:** 10.1038/s41598-017-08191-8

**Published:** 2017-08-11

**Authors:** Futoshi Toyoda, Pietro Mesirca, Stefan Dubel, Wei-Guang Ding, Joerg Striessnig, Matteo E. Mangoni, Hiroshi Matsuura

**Affiliations:** 1 Department of Physiology, Shiga University of Medical Science, Otsu Seta-Tsukinowa, Shiga, 520-2192 Japan; 2 CNRS, UMR-5203, Institut de Génomique Fonctionnelle, Département de Physiologie, LabEx ICST, Montpellier, F-34094 France; 3 INSERM, 1191, Montpellier, F-34094 France; 4 Université de Montpellier, UMR-5203, Montpellier, F-34094 France; 5 Department of Pharmacology and Toxicology, Institute of Pharmacy, Center for Molecular Biosciences, University of Innsbruck, Innsbruck, Austria

## Abstract

The spontaneous activity of sinoatrial node (SAN) pacemaker cells is generated by a functional interplay between the activity of ionic currents of the plasma membrane and intracellular Ca^2+^ dynamics. The molecular correlate of a dihydropyridine (DHP)-sensitive sustained inward Na^+^ current (*I*_st_), a key player in SAN automaticity, is still unknown. Here we show that *I*_st_ and the L-type Ca^2+^ current (*I*_Ca,L_) share Ca_V_1.3 as a common molecular determinant. Patch-clamp recordings of mouse SAN cells showed that *I*_st_ is activated in the diastolic depolarization range, and displays Na^+^ permeability and minimal inactivation and sensitivity to *I*_Ca,L_ activators and blockers. Both Ca_V_1.3-mediated *I*_Ca,L_ and *I*_st_ were abolished in Ca_V_1.3-deficient (Ca_V_1.3^−/−^) SAN cells but the Ca_V_1.2-mediated *I*_Ca,L_ current component was preserved. In SAN cells isolated from mice expressing DHP-insensitive Ca_V_1.2 channels (Ca_V_1.2^DHP−/−^), *I*_st_ and Ca_V_1.3-mediated *I*_Ca,L_ displayed overlapping sensitivity and concentration–response relationships to the DHP blocker nifedipine. Consistent with the hypothesis that Ca_V_1.3 rather than Ca_V_1.2 underlies *I*_st_, a considerable fraction of *I*_Ca,L_ was resistant to nifedipine inhibition in Ca_V_1.2^DHP−/−^ SAN cells. These findings identify Ca_V_1.3 channels as essential molecular components of the voltage-dependent, DHP-sensitive *I*_st_ Na^+^ current in the SAN.

## Introduction

Heart automaticity is generated by the spontaneous excitation of sinoatrial node (SAN) pacemaker cells. Spontaneous activity is due to the presence of the diastolic depolarization, which leads the membrane voltage from the end of the repolarization phase to the threshold of the following action potential. There has long been considerable debate regarding the ionic mechanisms underlying diastolic depolarization, reflecting the complex nature of this physiological process. Diastolic depolarization requires a net inward current, which results from the relative balance between the decaying outward delayed rectifier K^+^ currents (*I*_Kr_ and *I*_Ks_) and the growing inward currents (see Mangoni and Nargeot for review^1^). Previous studies have identified several voltage-gated inward currents activated in the diastolic depolarization range, including the hyperpolarization-activated inward current (*I*_f_)^2^, the L- and T-type Ca^2+^ currents (*I*_Ca,L_ and *I*_Ca,T_)^3, 4^ and the sustained inward Na^+^ current (*I*_st_)^5^. Additionally, recent experimental evidence has supported an alternative mechanism to promote pacemaker activity, in which spontaneous local Ca^2+^ release from intracellular Ca^2+^ stores stimulates electrogenic Na^+^–Ca^2+^ exchanger (*I*_NCX_) activity to depolarize the membrane voltage during diastolic depolarization^6^. Thus, multiple inward current systems rather than a single pacemaker current are responsible for the spontaneous activity in the SAN.

Selective pharmacological block or genetic ablation of ion channels has been extensively used to describe the contribution of ionic currents to pacemaker activity. The molecular correlates of most cardiac ionic currents have been identified allowing the development of genetically modified mouse models targeting specific ion channels including HCN4-^7, 8^, HCN2-*I*_f_^9, 10^, Ca_V_1.3-*I*_Ca,L_^11, 12^, Ca_V_3.1-*I*_Ca,T_^13^ and Ncx1-*I*_NCX_^14^. By contrast, the complete lack of knowledge about the molecular determinants of *I*_st_ has so far prevented the evaluation of the physiological role of this important ionic current.

*I*_st_ was reported as a novel inward current in SAN cells of several mammalian species including rabbits, guinea-pigs, rats and mice^5, 15–18^. *I*_st_ is activated at low membrane voltages and supplies persistent inward current flowing over the full diastolic depolarization range. Therefore, it has been proposed that this current is a physiologically important contributor to diastolic depolarization^19^. However, two decades after the first description of *I*_st_ by Guo *et al*.^5^, there remains little progress in the identification of the molecular determinant of *I*_st_. Furthermore, no specific blocker for *I*_st_ is available, limiting the understanding of its physiological role in SAN pacemaker activity. Although *I*_st_ is carried by Na^+^, its pharmacological features closely resemble those of *I*_Ca,L_: *I*_st_ is not affected by the voltage-gated Na^+^ current (*I*_Na_) blocker tetrodotoxin (TTX), but is inhibited by various chemical classes of organic Ca^2+^ channel blockers, e.g. dihydropyridines (DHPs)^5^, and enhanced by the *I*_Ca,L_ channel activator Bay-K8644^15^. Moreover, like *I*_Ca,L_, *I*_st_ is also stimulated by β-adrenergic activation^5, 18^. These pharmacological properties are highly specific for *I*_Ca,L_ and suggest the possibility that the pore-forming α_1_-subunit of L-type Ca^2+^ channels which carry the drug-binding domains for organic Ca^2+^-channel blockers and activators^20, 21^ are also essential for *I*_st_ activity. Cardiac L-type Ca^2+^ channels are heteromultimers in which the pore-forming α_1_ subunit associates with auxiliary subunits (in particular β, α_2_/δ subunits)^22^. In SAN cells, two different α_1_-subunits, Ca_V_1.2 (α_1C_) and Ca_V_1.3 (α_1D_), are expressed. Ca_V_1.2 is uniformly expressed in heart tissue, whereas Ca_V_1.3 is nearly absent in ventricles but is abundant in the conduction system including the SAN^12, 23, 24^. While Ca_V_1.3 channels activate in the diastolic depolarization range, Ca_V_1.2 channels are activated in the upstroke phase of the action potential^12^. In addition to forming distinct types of *I*_Ca,L_ with different voltage dependencies of activation and inactivation, Ca_V_1.3 and Ca_V_1.2 channels are also differentially localized in SAN cell membranes^25^.

Here we tested the hypothesis that Ca_V_1.2 and/or Ca_V_1.3 L-type channels are required for generating *I*_st_. Using two genetically modified mouse strains we demonstrate that the Ca_V_1.3 L-type Ca^2+^-channel isoform is essential for functional expression of *I*_st_ in mouse SAN cells. Although the exact molecular mechanism linking Ca_V_1.3 activity to *I*_st_ remains to be elucidated, our data show that Ca_V_1.3 channels participate in the formation of a DHP-sensitive, voltage-dependent Na^+^ conductance in SAN cells.

## Results

### Identification of ***I***_**st**_ in mouse SAN cells

**Figure 1:**
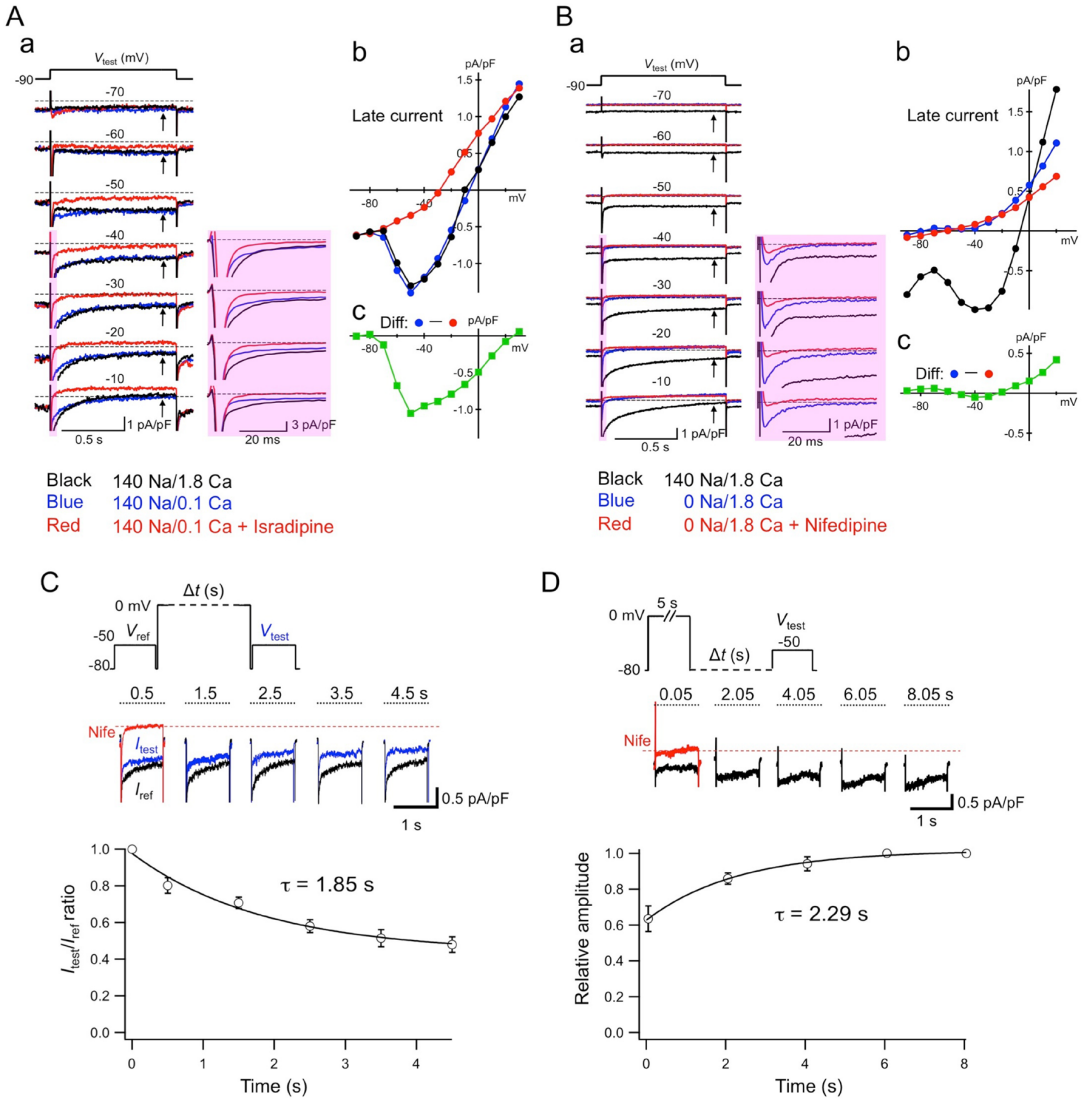
Presence of *I*_st_ in mouse SAN cells. (**A**) (a) Superimposed whole-cell membrane currents recorded from the same cell in 1.8 mM [Ca^2+^]_o_ (control, *black*), in 0.1 mM [Ca^2+^]_o_ (*blue*) and after exposure to 1 µM isradipine (*red*). Individual SAN cells were voltage clamped at a holding potential of −90 mV and depolarized for 1 s to indicated test potentials in 10-mV increments. Peaks of transient inward currents at the beginning of test pulses are not to scale. The *inset* shows close-up views of the initial part (*red shaded area*) of current traces. (**b**) Corresponding isochronal *I*–*V* relationships of the late current measured at time points marked with arrows in a. (c) *I*–*V* relationship of the isradipine-sensitive current obtained by subtraction of the current recorded after application of isradipine from that recorded in 0.1 mM [Ca^2+^]_o_. (**B**) (a) Superimposed whole-cell membrane currents recorded in the same cell in 1.8 mM [Ca^2+^]_o_ (control, *black*), in NMDG-substituted, Na^+^-free solution (*blue*) and after exposure to 1 µM nifedipine (*red*) using the same pulse protocol as in (**A**). The *inset* shows close-up views of the initial part (*red shaded area*) of current traces. (b) Corresponding *I*–*V* relationships of the late currents from the recording depicted in a. (c) *I*–*V* relationship of the nifedipine-sensitive current obtained by subtraction of currents after application of nifedipine from recordings in the NMDG-substituted, Na^+^-free solution. (**C**) Time dependency of the sustained inward current inactivation measured using a protocol (*upper panel*) consisting of a reference test pulse (*V*_ref_), a conditioning prepulse of various durations, and a subsequent test pulse (*V*_test_). Sample traces of *I*_ref_ (*black*) and *I*_test_ (*blue*) recorded in response to *V*_ref_ and *V*_test_, respectively, in 0.1 mM [Ca^2+^]_o_ were superimposed. The *red* trace (Nife) was recorded in the presence of 1 µM nifedipine and indicates the background level at −50 mV (*red dash line*). The dotted line (*black*) indicates the zero-current level. The *bottom panel* shows a plot of the average ratio of *I*_test_/*I*_ref_ as a function of the conditioning pulse duration. The continuous line represents a single exponential fit. (**D**) Time dependency of the recovery from inactivation of the sustained inward current measured using a double-pulse protocol (*upper panel*) with varying recovery intervals (0.05–8.05 s) at −80 mV between a 5-s conditioning prepulse to 0 mV and a test pulse (*V*_test_) to −50 mV. Current amplitudes are normalized to the largest current obtained with a recovery interval of 8.05 s. Sample traces and panels are labelled as in (**C**).

The magnitude of *I*_st_ varies depending on SAN cell types with distinct morphologies^5^. Mouse SAN cells used for *I*_st_ recordings were typically spindle- or spider-shaped with no obvious striations. These cells were small (*C*_m_, 34.8 ± 1.2 pF, n = 42) compared to rod-shaped atrial-like cells and were spontaneously beating when superfused with normal Tyrode solution. To confirm the presence of *I*_st_ in these cells, the late currents elicited by 1-s depolarizing voltage-clamp steps to various test potentials from a holding potential of −90 mV were examined for the characteristics of *I*_st_ (Fig. 1). In order to avoid contamination of recordings by K^+^ currents, we employed a Cs^+^-rich internal solution. *I*_f_ was removed by substituting K^+^ with Cs^+^ in the external Tyrode solution, which contained 1.8 mM Ca^2+^. To confirm the sensitivity of the sustained current to DHPs, the typical hallmark of *I*_st_^19^, we tested the sensitivity of the current to the potent DHP L-type Ca^2+^-channel blocker isradipine, which has not previously been tested. In Fig. 1Aa, membrane currents recorded under control conditions (*black trace*), after lowering [Ca^2+^]_o_ from 1.8 to 0.1 mM (*blue trace*) and during subsequent application of 1 µM isradipine (*red trace*) are superimposed at individual test potentials. In the control bathing solution, membrane depolarization positive to −60 mV evoked a large transient inward current attributable to the activation of *I*_Na_ and *I*_Ca,T_ (note that peaks are not to scale in the figure), followed by a late inward current sustained during the entire period of 1-s depolarizing pulses. An inward current with a slow current decay was observed at test potentials of >−40 mV, as expected for *I*_Ca,L_ activation. The current-to-voltage (*I*–*V*) relationship obtained by plotting the current amplitude measured near the end of test pulses indicated that the late current level becomes more inward with increasing depolarization between −70 and −50 mV (*black circles*, Fig. 1Ab), generating a negative slope conductance in the range of the diastolic depolarization. Lowering external Ca^2+^ reduced a considerable fraction of *I*_Ca,L_ at membrane voltages positive to −30 mV (*inset*, Fig. 1Aa), whereas the sustained inward current was not reduced. It is thus unlikely that the sustained inward current was generated by a window component of *I*_Ca,L_. However, bath application of isradipine readily inhibited the sustained inward current and unmasked an almost linear background conductance (Fig. 1Aa,b). Under conditions of low [Ca^2+^]_o_, the DHP-sensitive sustained inward current peaked at −50 mV and the current direction was reversed at ~+ 26 mV (Fig. 1Ac).

Since *I*_st_ has been shown to be carried by Na^+^^5^, we tested the permeability of the sustained inward current component for Na^+^. The external Na^+^ was replaced with an equimolar amount of N-methyl-D-glucamine (NMDG) in the presence of 1.8 mM Ca^2+^ (Fig. 1B). Perfusion of SAN cells with Na^+^-free NMDG solution readily suppressed the sustained inward current as well as Na^+^-dependent background conductance (Fig. 1Ba and b). Subsequent application of 1 µM nifedipine did not affect the late inward current component, indicating that Na^+^ was the predominant ion carrying the DHP-sensitive sustained inward current. At voltages positive to −20 mV the outward current component was partially reduced by nifedipine (average current density of the DHP-sensitive outward current, 0.34 ± 0.08 pA/pF at + 20 mV; n = 4, two independent experiments: N = 2), suggesting that Cs^+^ was carrying the DHP-sensitive current component.

We next evaluated the kinetics of inactivation of the sustained inward current in mouse SAN cells in further detail (Fig. 1C and D). In the experiment shown in Fig. 1C, the inactivation time course was determined by measuring the fractional change of the sustained current elicited by a depolarizing step to −50 mV from a holding potential of −80 mV in 0.1 mM [Ca^2+^]_o_, immediately (0.05 s) before (*I*_ref_) and after (*I*_test_) conditioning pulses to 0 mV of variable duration (0.5–4.5 s). Nifedipine was then applied to acquire the background current (*red trace*) at −50 mV, which was used to evaluate the net amplitude of the DHP-sensitive inward current. In Fig. 1C the ratio of *I*_test_/*I*_ref_ is plotted as a function of the conditioning pulse duration, indicating that while *I*_st_ displayed slow inactivation (τ = 1.94 ± 0.57s, n = 3, N = 1), a considerable current fraction remained available even after a 4.5-s conditioning pulse (0.48 ± 0.04, n = 3, N = 1). In addition, recovery from inactivation was assessed by applying a 5-s conditioning prepulse followed by test pulses to −50 mV after varying intervals of recovery (0.05–8.05 s) at −80 mV (Fig. 1D). Recovery of the sustained current proceeded exponentially with a time constant of 2.66 ± 0.63s (n = 3, N = 1).

These properties (low voltage for activation, DHP sensitivity, Na^+^ permeability and slow inactivation), clearly identified the sustained inward current in our mouse SAN cell preparations as *I*_st_^5, 15–18, 26^. We only failed to record the sustained current in five of 24 experiments (~20%), which is likely to indicate inhomogeneous expression of *I*_st_ in SAN cells^16^. Four of five *I*_st_-deficient cells were nearly indistinguishable from clear striated atrial-like myocytes.

### ***I***_**Na**_ and ***I***_**NCX**_ do not contribute to ***I***_**st**_ in mouse SAN

**Figure 2:**
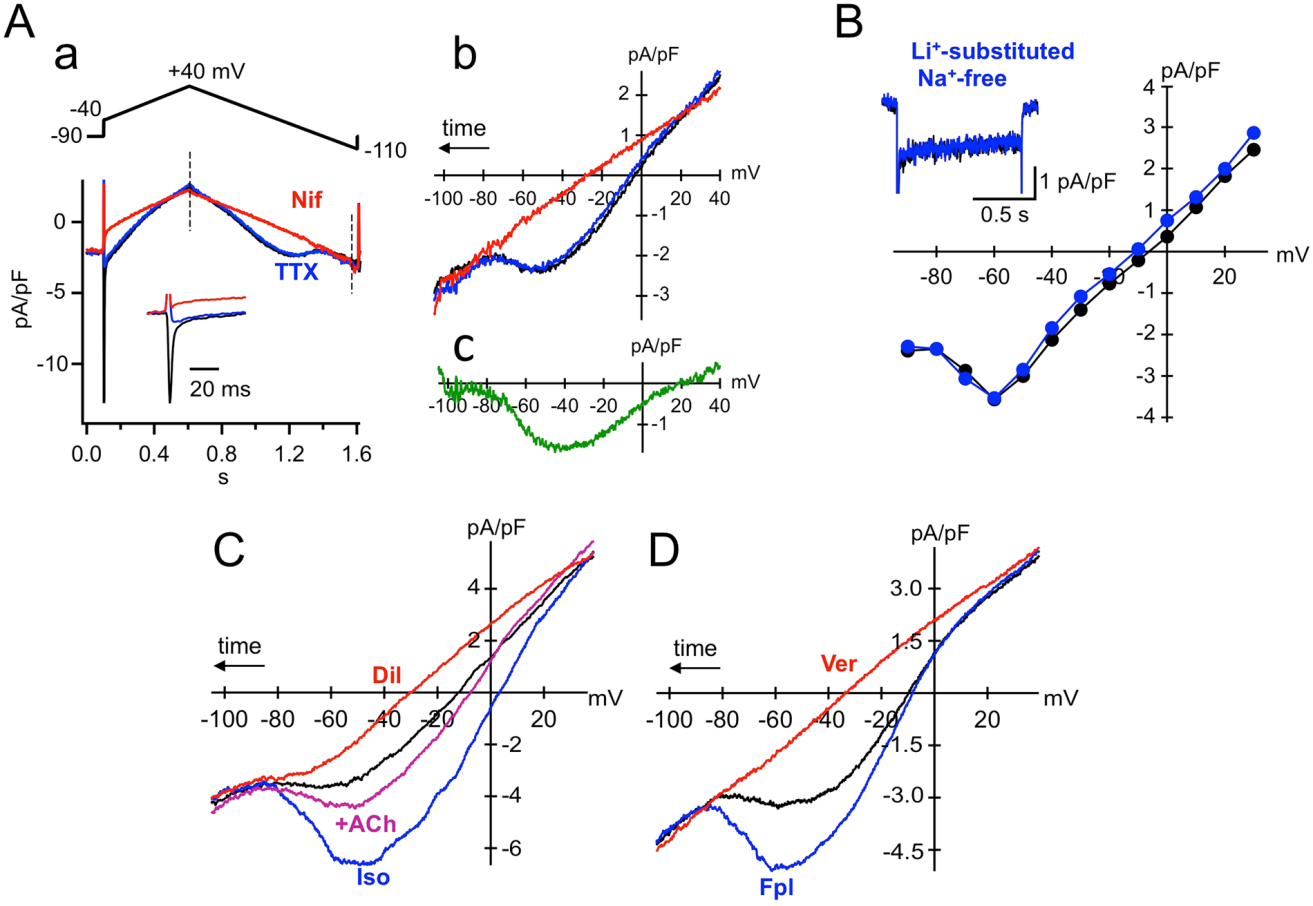
Pharmacological properties of *I*_st_ in mouse SAN cells. (**A**) Effects of TTX on *I*_st_. (a) Voltage ramp pulse protocol and original current traces recorded from the same cell in 0.1 mM [Ca^2+^]_o_ (control, *black*), during exposure to 10 µM TTX (*blue*) and after subsequent addition of 1 µM nifedipine (*red*). The inset shows expanded traces at the beginning of the voltage-command pulse. (b) Corresponding *I*–*V* relationship obtained from current recordings during the descending ramp from + 40 to −110 mV in a. (c) *I*–*V* relationship of *I*_st_ isolated by subtracting current recordings before and after application of nifedipine in the presence of TTX. (**B**) Effects of Na^+^ replacement with Li^+^ on *I*_st_. The *I*–*V* relationships were constructed on current recordings before (*black*) and after (*blue*) total replacement of Na^+^ with Li^+^ in the 0.1 mM [Ca^2+^]_o_ solution. Currents were elicited by 1-s depolarizing pulses (10 mV increment) to various test potentials from a holding potential of −90 mV. The inset shows original current traces at −50 mV. (**C**) Effects of autonomic agonists on *I*_st_. The *I*–*V* relationships were obtained from current recordings in the same cell during the descending limb of a voltage ramp (similar to (**A**)) in the control 0.1 mM [Ca^2+^]_o_ solution (*black*), during exposure to 100 nM Iso (*blue*) and Iso plus 1 µM ACh (*purple*), and after addition of 1 µM diltiazem (Dil, *red*). (**D**) Effects of a non-DHP *I*_Ca,L_ agonist on *I*_st_. Superimposed *I*–*V* relationships were obtained during the descending limb of a voltage ramp (similar to (**A**)) in the control 0.1 mM [Ca^2+^]_o_ solution (*black*), during exposure to 1 µM FPL-64176 (Fpl, *blue*) and after subsequent addition of 1 µM verapamil (Ver, *red*).

To assess whether voltage-gated Na^+^ currents could interfere with *I*_st_ recordings in mouse SAN cells, we investigated the effect of the *I*_Na_ blocker TTX on the membrane current (Fig. 2A). Since *I*_st_ exhibited little inactivation during the 1-s square pulse, the *I*–*V* relationship was measured using a slow (150 mV/s) voltage-ramp protocol in 0.1 mM [Ca^2+^]_o_. Under these conditions, the contributions of *I*_Ca,L_ and *I*_Ca,T_ to the total membrane current were minimized^5, 15, 18^. Figure 2Aa shows a superimposition of the original current traces in response to the voltage ramp in the control (*black trace*), during 10 µM TTX application (*blue trace*) and after nifedipine application (*red trace*). Figure 2Ab displays the corresponding *I*–*V* relationships obtained from the descending limb of the voltage ramp. As evidenced in the current recordings and in the corresponding *I*–*V* curve, bath application of TTX readily inhibited the transient inward *I*_Na_ at the beginning of the pulse (see *expanded traces in the inset*), but did not affect the subsequent current. Application of nifedipine (1 µM) then revealed that the TTX-insensitive and DHP-sensitive current component could be attributed to *I*_st_ (Fig. 2Ac). It should be noted that partial inhibition of the late inward current by TTX was observed in six of 23 cells (26%) (average current density of TTX-sensitive current, 0.82 ± 0.17 pA/pF at −50 mV; n = 6, N = 4), suggesting that some mouse SAN cells also express a TTX-sensitive persistent Na^+^ current^27^.

The involvement of *I*_NCX_ was also investigated (Fig. 2B). *I*_st_ was hardly affected by total replacement of external Na^+^ with an equimolar concentration of Li^+^ to abolish *I*_NCX_. This result is consistent with the previously characterized selectivity of *I*_st_ to monovalent cations^26^. In conclusion, *I*_NCX_ did not contaminate our recordings of *I*_st_.

### Sensitivity of ***I***_**st**_ to ***I***_**Ca,L**_ modulators in mouse SAN cells

We then characterized the pharmacological properties of *I*_st_ by testing its sensitivity to various *I*_Ca,L_ modulators in the presence of 0.1 mM [Ca^2+^]_o_ solution. In the experiment shown in Fig. 2C, 0.1 µM isoprenaline (Iso) strongly increased *I*_st_ (116.1 ± 16.8%, n = 8, N = 2, p = 0.0003). This stimulatory effect was almost reversed by addition of 1 µM acetylcholine (ACh) in the presence of Iso (84.3 ± 2.7%, n = 4, N = 2, p = 0.0029). Finally, the non-DHP *I*_Ca,L_ blocker diltiazem (1 µM) completely abolished *I*_st_. By contrast, the *I*_Ca,L_ agonist FPL-64176 (1 µM) potentiated the amplitude of *I*_st_ nearly twofold, whereas application of 1 µM verapamil totally abolished *I*_st_ (Fig. 2D). Our observations, in addition to the findings of previous studies^5, 15, 18^, indicate that the pharmacological properties of *I*_st_ are undistinguishable from those of *I*_Ca,L_ (Table 1).

**Table 1:** Pharmacological similarities between *I*_st_ and *I*_Ca,L_.

Drugs	Concentration [μM]	*I*_st_	*I*_Ca,L_	References
**Dihydropyridines**				
Nicardpine	0.25–0.5	block	block	Guo *et al*.^5^
Nifedipine	0.03–1.0	block	block	this study
Isradipine	1.0	block	block	this study
Bay-K8644 (agonist)	1.0	increase	increase	Guo *et al*.^15^
**Benzothiazepines**				
Diltiazem	1.0	block	block	this study
**Phenylalkylamines**				
D600	0.1	block	block	Guo *et al*.^5^
Verapamil	1.0	block	block	this study
**FPL-64176 (agonist)**	1.0	increase	increase	this study
**Tetrodotoxin**	10–30	no effect	no effect	Guo *et al*.^5^; this study
**Isoprenaline**	~0.1	increase	increase	Toyoda *et al*.^18^; this study

### ***I***_**st**_ is absent in Ca_V_1.3^−/−^ SAN cells

**Figure 3:**
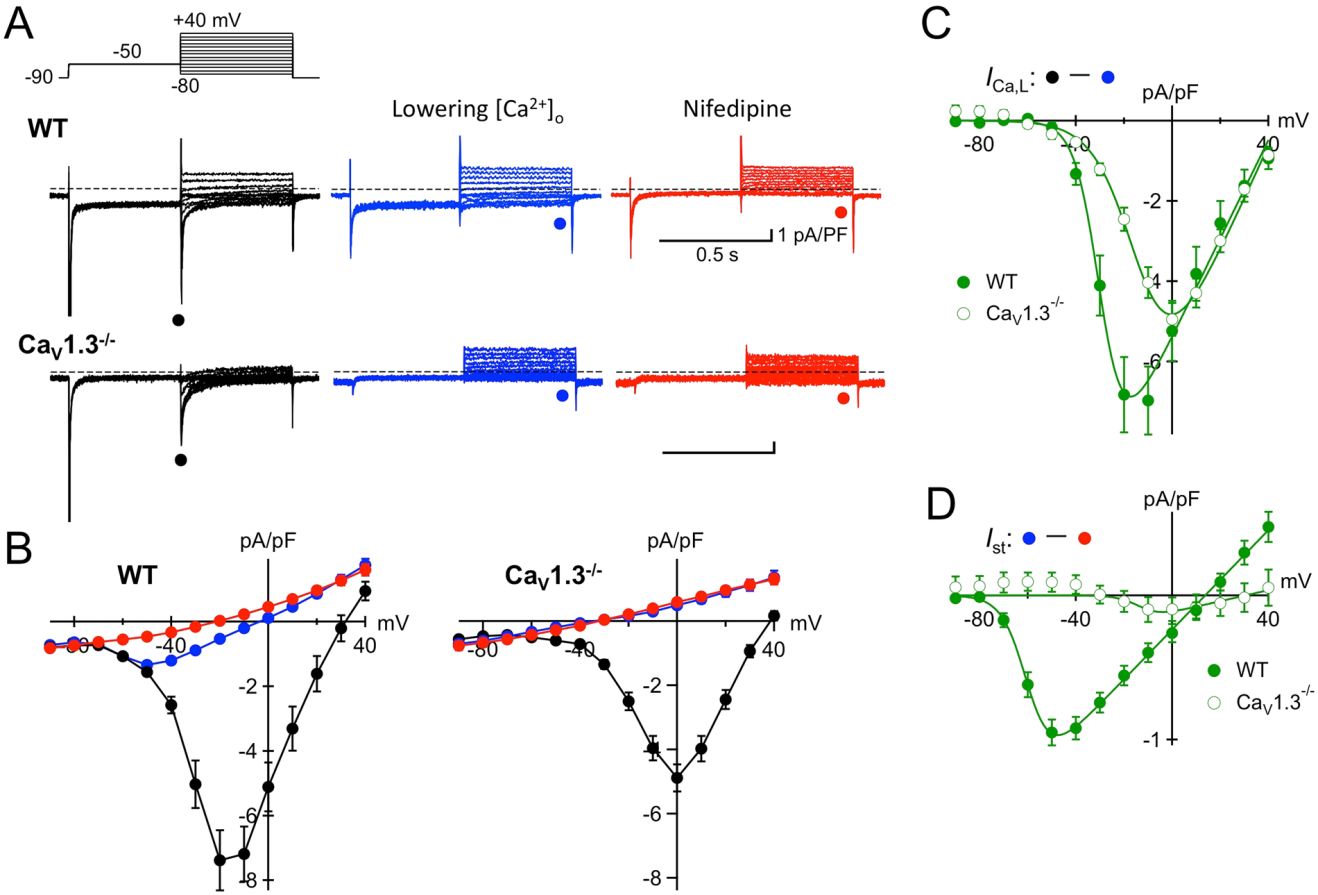
Absence of *I*_st_ in SAN cells from Ca_V_1.3^−/−^ mice. (**A**) Representative examples of current recordings in SAN cells obtained from wild-type (*upper panel*) and Ca_V_1.3^−/−^ (*lower panel*) mice. Currents were elicited by voltage steps to test potentials between −80 and + 40 mV (10 mV increments) preceded by a conditioning pulse to −50 mV from a holding potential of −90 mV in 1.8 mM [Ca^2+^]_o_ (control, *black*), in 0.1 mM [Ca^2+^]_o_ solution containing 10 µM TTX (*blue*) and after applying 1 µM nifedipine (*red*). (**B**) Average *I*–*V* relationships of current densities measured at the time points indicated in (**A**). Data represent the mean ± S.E.M. of wild-type (*left*, n = 19) and Ca_V_1.3^−/−^ (*right*, n = 18) SAN cells. (**C**), *I*–*V* relationships of *I*_Ca,L_ in wild-type (*closed symbols*) and Ca_V_1.3^−/−^ (*open symbols*) SAN cells, obtained by subtraction of currents after lowering [Ca^2+^]_o_ from recordings in the control solution. (**D**) *I*–*V* relationships of *I*_st_ in wild-type (*closed symbols*) and Ca_V_1.3^−/−^ (*open symbols*) SAN cells, measured as the nifedipine-sensitive current in 0.1 mM [Ca^2+^]_o_.

The undistinguishable pharmacological properties of *I*_st_ and *I*_Ca,L_ (Table 1) provided a strong rationale for testing the hypothesis that these currents share common molecular determinants. It is now generally accepted that *I*_Ca,L_ in SAN cells is composed of two separate current components mediated by distinct pore-forming alpha subunits, Ca_V_1.2 and Ca_V_1.3^11, 12^. To directly examine the possibility of a functional link between *I*_st_ and Ca_V_1.3, we recorded *I*_Ca,L_ and *I*_st_ in SAN cells from mice lacking Ca_V_1.3 channels (Ca_V_1.3^−/−^ mice, Fig. 3). Since most SAN cells obtained from Ca_V_1.3^−/−^ mice were quiescent, we selected single cells for recordings based on morphological criteria rather than spontaneous activity. After the control recording in Cs^+^-substituted, K^+^-free Tyrode solution with 1.8 mM [Ca^2+^]_o_ (*black traces*), *I*_st_ was separated from *I*_Ca,L_ by switching the bath solution to 0.1 mM [Ca^2+^]_o_ containing 10 µM TTX (*blue traces*). *I*_st_ was identified as a current component inhibited by subsequent application of 1 µM nifedipine (*red traces*). Consistent with previous studies, genetic ablation of Ca_V_1.3 channels resulted in considerable reduction of *I*_Ca,L_^12^ as well as a shift in the current half-activation voltage^11, 12^ (Fig. 3A–C). Indeed, the peak density of *I*_Ca,L_ was significantly reduced from −6.97 ± 0.85 pA/pF in wild-type SAN cells (n = 19, N = 6) to −4.81 ± 0.45 pA/pF in Ca_V_1.3^−/−^ cells (n = 18, N = 6, p = 0.0336), and was accompanied by a positive shift in the peak of the *I–V* relationship by ~20 mV (Fig. 3C). The calculated half-maximal activation voltage (*V*_0.5act_) was shifted from −29.3 mV in wild-type cells to −12.8 mV in Ca_V_1.3^−/−^ SAN cells.

*I*_st_ was evident in wild-type SAN cells after *I*_Ca,L_ removal by lowering [Ca^2+^]_o_, as manifested by the increase in the sustained inward current with depolarization between −70 and −50 mV that was finally blocked by nifedipine (Fig. 3A and B). By contrast, the late current obtained from Ca_V_1.3^−/−^ SAN cells changed linearly with command voltage in the 0.1 mM [Ca^2+^]_o_ solution with 10 µM TTX, and there remained no detectable DHP-sensitive current. As shown in Fig. 3D, the average peak density of *I*_st_ was −0.98 ± 0.09 pA/pF (n = 19, N = 6) in wild-type cells, while it was reduced below detectable levels in Ca_V_1.3^−/−^ cells (n = 18, N = 6). Thus, we concluded that *I*_st_ was virtually absent in SAN cells from Ca_V_1.3^−/−^ mice.

**Figure 4:**
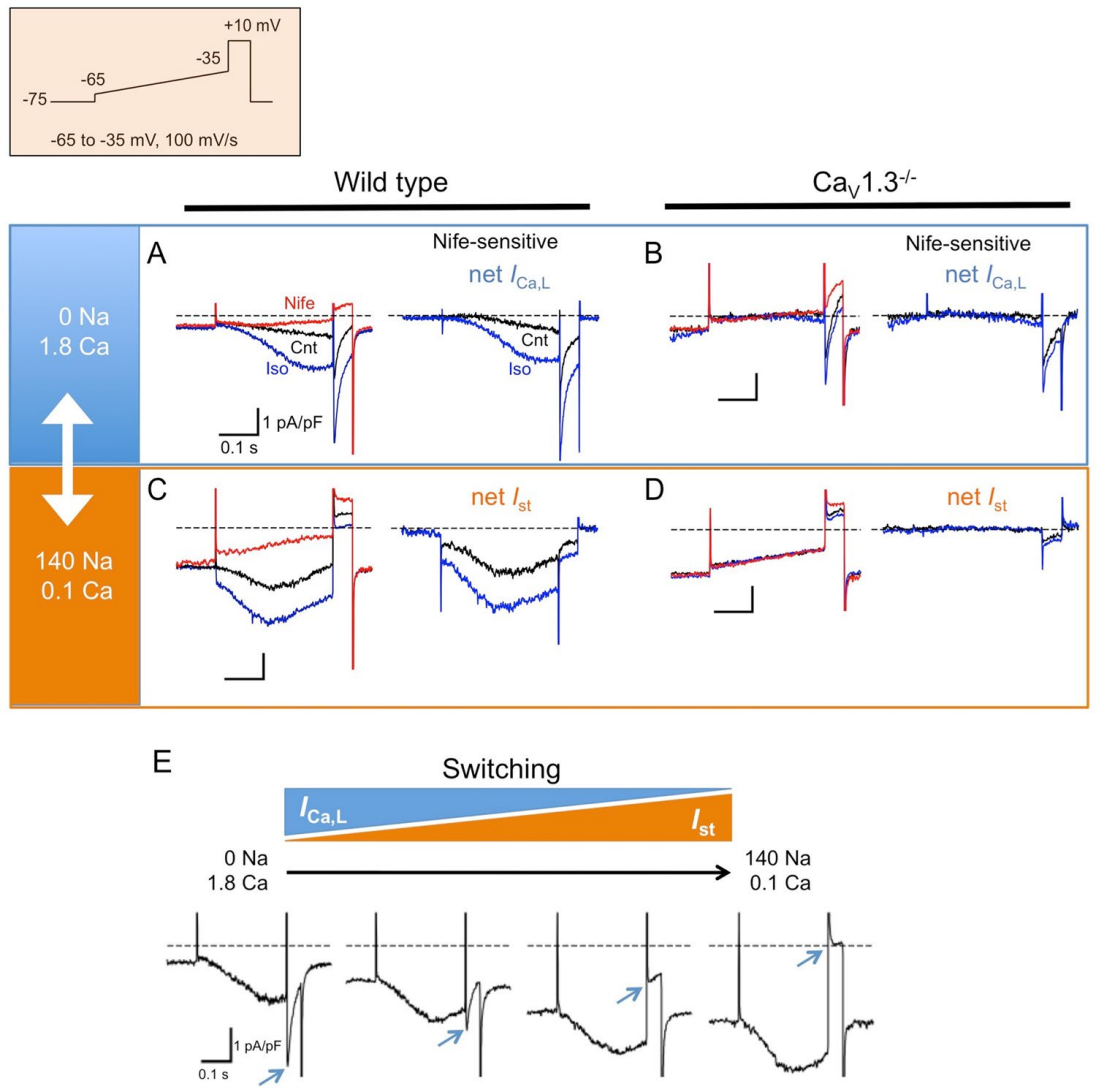
Ca_V_1.3 mediates both *I*_Ca,L_ and *I*_st_ in the SAN diastolic depolarization range. (**A**,**C**) Representative whole-cell membrane currents recorded from the same wild-type SAN cell using two different external solutions: TEA^+^-substituted, Na^+^-free external solution containing 1.8 mM Ca^2+^ (**A**) to record *I*_Ca,L_ and 140 mM [Na^+^]_o_ solution containing 0.1 mM Ca^2+^ plus 10 µM TTX (**C**) to record *I*_st_. The cell was first held at −75 mV. Then, a slow ascending ramp (100 mV/s) voltage command was used to elicit *I*_Ca,L_ or *I*_st_ (*top panel*), from −65 to −35 mV, followed by depolarization to +10 mV for 50 ms. Under distinct external conditions, currents were recorded in the absence (*black trace, left panel*) and presence (*blue trace, left panel*) of 100 nM Iso, and after addition of 1 µM nifedipine (*red trace, left panel*). Nifedipine-sensitive net *I*_Ca,L_ and *I*_st_ in the absence (*black trace, right panel*) and presence (*blue trace, right panel*) of Iso were obtained by digital subtraction of current traces before and after application of nifedipine (*right panel*). (**B**,**D**) Representative *I*_Ca,L_ (**B**) and *I*_st_ (**D**) in Ca_V_1.3^−/−^ SAN cells, recorded using the same protocol as in (**A**,**C**), respectively. (**E**) Sample current recordings in a wild-type SAN cell during gradual replacement of the external solution for *I*_Ca,L_ recording with that for *I*_st_ recording in the presence of Iso. Arrows indicate the peak of *I*_Ca,L_.

The above results suggested that Ca_V_1.3 mediated two different currents in SAN cells, i.e. Ca^2+^-conducting *I*_Ca,L_ and Na^+^-conducting *I*_st_. To support this hypothesis and estimate the contribution of Ca_V_1.3-mediated *I*_Ca,L_ and *I*_st_ to the diastolic depolarization, *I*_Ca,L_ and *I*_st_ were alternately recorded in the same cell under distinct external ionic conditions and activation in the diastolic depolarization range was evaluated in wild-type and Ca_V_1.3^−/−^ SAN cells (Fig. 4). A slow ascending ramp (−65 to −35 mV, 100 mV/s) voltage command was employed to mimic the diastolic depolarization. We first recorded *I*_Ca,L_ using the 0 Na^+^, 1.8 mM Ca^2+^ external solution (Fig. 4A). Under these recording conditions, the voltage ramp gradually activated an inward current yielding negative slope conductance in wild-type SAN cells. This current was strongly augmented by Iso and inhibited by subsequent application of nifedipine. The nifedipine-sensitive difference current showed that the net *I*_Ca,L_ started to activate clearly within the diastolic depolarization range, as expected for the low-voltage activation of Ca_V_1.3-mediated *I*_Ca,L_. The average threshold for activation of Ca_V_1.3-mediated *I*_Ca,L_ was −51.2 ± 1.0 mV under control conditions and −59.8 ± 0.9 mV upon perfusion of Iso (n = 7, N = 3). Iso significantly augmented the amount of charge carried by Ca_V_1.3-mediated *I*_Ca,L_ from 0.068 ± 0.013 to 0.177 ± 0.023 pQ/pF (n = 7, N = 3, p = 0.0003). In contrast to wild-type SAN cells, significant nifedipine-sensitive Ca_v_1.3-mediated *I*_Ca,L_ was not recorded in Ca_v_1.3^−/−^ SAN cells (Fig. 4B). Similar to wild-type cells, Ca_V_1.2-mediated *I*_Ca,L_ could be elicited by subsequent depolarization at +10 mV. We did not find a statistically significant difference in the response of *I*_Ca,L_ at +10 mV to Iso between wild-type (112.0 ± 8.7%, n = 7, N = 3) and Ca_V_1.3^−/−^ SAN cells (94.6 ± 6.1%, n = 6, N = 3, p = 0.1319). Taken together, these observations indicated that Ca_V_1.3 channels alone fully accounted for *I*_Ca,L_ in the pacemaker potential range.

We then switched to an external recording solution containing 140 mM Na^+^, 0.1 mM Ca^2+^ and 10 µM TTX to record *I*_st_ in the same cells. As illustrated in Fig. 4E, slow replacement of the bathing solution enabled the monitoring of gradual changes in membrane currents. These changes included a marked inward shift in the holding current and a reduction in *I*_Ca,L_ at +10 mV (indicated by arrows). In contrast to *I*_Ca,L_, we observed an increase in the inward current accompanied by a negative shift in the peak potential, which indicated that increased *I*_st_ offsets the loss of *I*_Ca,L_ along the voltage ramp. Similar to *I*_Ca,L_, *I*_st_ was enhanced by Iso and blocked by nifedipine (Fig. 4C). *I*_st_ was detected in all wild-type SAN cells (0.228 ± 0.039 and 0.453 ± 0.076 pQ/pF in the absence and presence of Iso, respectively, n = 7, N = 3). However, we failed to record *I*_st_ in Ca_V_1.3^−/−^ SAN cells (0.012 ± 0.002 and 0.014 ± 0.004 pQ/pF in the absence and presence of Iso, respectively, n = 6, N = 3, Fig. 4D).

We did not observe either *I*_st_ or *I*_Ca,L_ in atrial-like myocytes isolated from wild-type SAN (n = 4, N = 3, data not shown). Thus, the presence of *I*_st_ was always coupled to the low voltage-activated *I*_Ca,L_, which is consistent with the view that Ca_V_1.3 mediates both *I*_Ca,L_ and *I*_st_.

### Ca_V_1.2 channels are not involved in the generation of ***I***_**st**_

**Figure 5:**
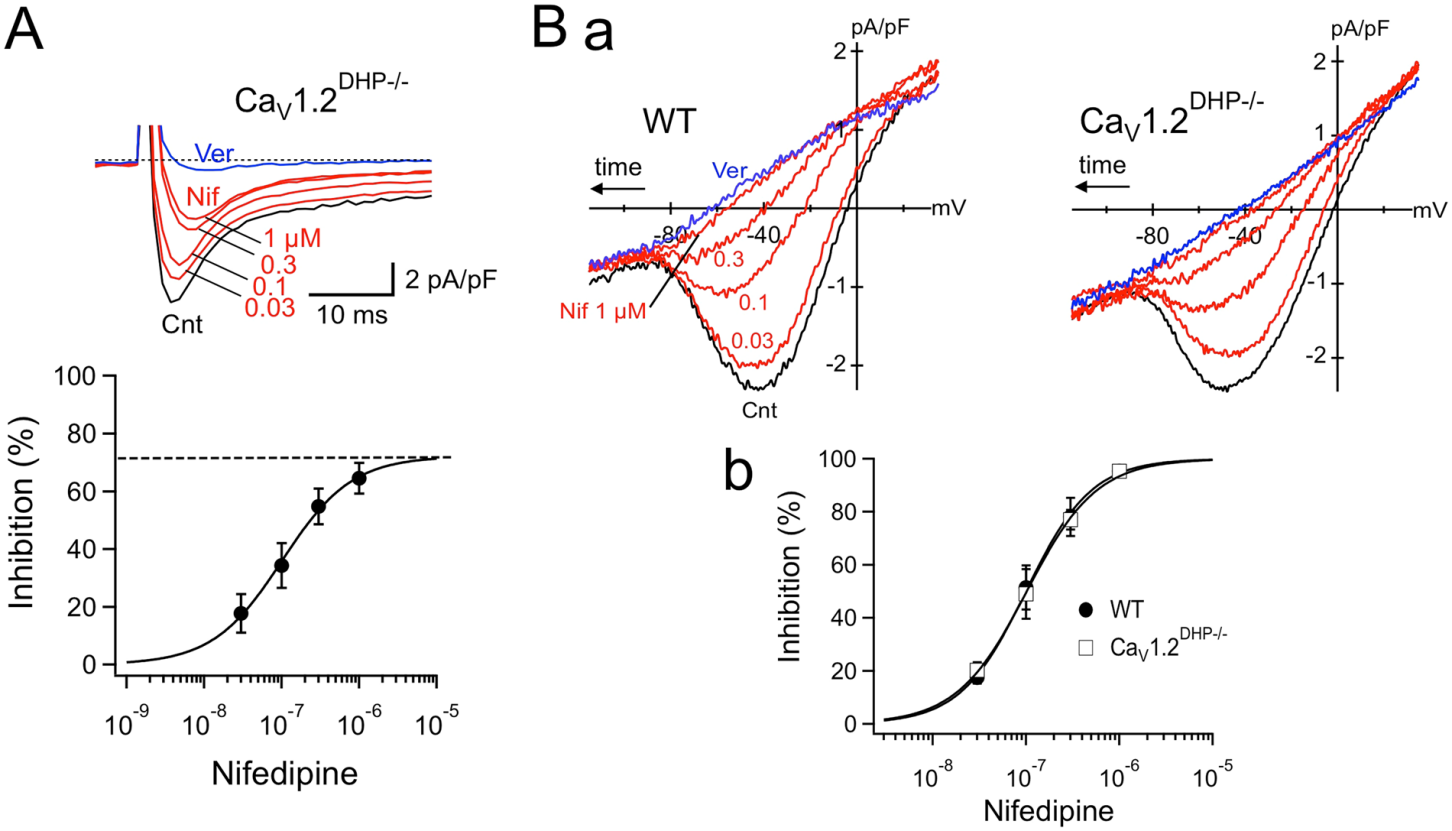
Sensitivity of *I*_Ca,L_ and *I*_st_ to nifedipine in SAN cells from Ca_V_1.2^DHP−/−^ mice. (**A**) *I*_Ca,L_ inhibition by nifedipine in Ca_V_1.2^DHP−/−^ SAN cells. (a) Superimposed sample traces of *I*_Ca,L_ elicited by depolarization to −10 mV from a holding potential of −50 mV in TEA^+^-substituted, Na^+^-free Tyrode solution (control, *black*) and during nifedipine application at various concentrations (0.03–1 µM, *red*), and after subsequent addition of 3 µM verapamil (*blue*). (b) Concentration-dependent inhibition of *I*_Ca,L_ by nifedipine. Inhibition is expressed as the percentage of residual *I*_Ca,L_ inhibited by nifedipine relative to control *I*_Ca,L_. Data are mean ± S.E.M. of four experiments. The smooth curve represents the least squares fit of data points using the Hill equation, yielding a maximal current response of 72%, IC_50_ of 101.7 nM and Hill coefficient of 0.97. (**B**) Effects of nifedipine on *I*_st_ in wild-type and Ca_V_1.2^DHP−/−^ SAN cells. (a) Superimposed *I*–*V* relationships of *I*_st_ obtained by voltage ramp in wild-type (*left*) and Ca_V_1.2^DHP−/−^ (*right*) SAN cells in the control 0.1 mM [Ca^2+^]_o_ solution (control, *black*), during exposure to various concentrations of nifedipine (0.03–1 µM, *red*), and after application of 3 µM verapamil (*blue*). (b) Concentration–response relationship of *I*_st_ inhibition by nifedipine in wild-type (*closed circles*) and Ca_V_1.2^DHP−/−^ (*open squares*) SAN cells. Data are mean ± S.E.M. of four independent measurements. The line represents the fit of the Hill equation.

The absence of *I*_st_ in Ca_V_1.3^−/−^ SAN cells does not exclude the possibility that Ca_V_1.2 also contributes to the generation of *I*_st_. To examine the involvement of Ca_V_1.2 in *I*_st_, we employed knock-in mice in which a point mutation (T1066Y) abolishes the sensitivity of Ca_V_1.2 to DHPs without changing channel function and expression (Ca_V_1.2^DHP−/−^ mice, Fig. 5)^28^. In this mouse model, selective blockade of Ca_V_1.3 by DHPs enables the functional contributions of Ca_V_1.2 and Ca_V_1.3 to the generation of *I*_Ca,L_ to be distinguished. We first tested the effect of nifedipine on *I*_Ca,L_ in SAN cells from Ca_V_1.2^DHP−/−^ mice (Fig. 5A). *I*_Ca,L_ was recorded after elimination of *I*_st_ and *I*_Na_ by Na^+^ removal from the external recording solution. Bath application of nifedipine (0.03–1 µM) reduced the peak amplitude of *I*_Ca,L_ in a concentration-dependent manner to a maximum of ~64% even at a saturating concentration of DHP (1 µM). This residual DHP-resistant *I*_Ca,L_ was completely blocked by application of verapamil (3 µM), in line with previous data showing that the T1066Y mutation preserves the high sensitivity of Ca_V_1.2 to phenylalkylamines^29^. Peak inward current of the nifedipine-resistant component activated more slowly (Fig. 5A), as expected for Ca_V_1.2-mediated *I*_Ca,L_^30^. The presence of this DHP-insensitive component is consistent with our earlier finding^11, 12^ that *I*_Ca,L_ in SAN cells is mediated by both Ca_V_1.2 and Ca_V_1.3.

We then examined whether altered sensitivity of Ca_V_1.2 to DHP also affected the response of *I*_st_ to nifedipine. The concentration-dependent inhibition of *I*_st_ by nifedipine was investigated in SAN cells isolated from wild-type and Ca_V_1.2^DHP−/−^ mice (Fig. 5B). After suppressing *I*_Ca,L_ by lowering [Ca^2+^]_o_ to 0.1 mM, *I*_st_ was elicited by the voltage ramp in the presence of nifedipine at various concentrations (0.03–1 µM). *I*_st_ in Ca_V_1.2^DHP−/−^ SAN cells was reduced by nifedipine to levels similar to those observed in wild-type cells. Indeed, we failed to detect a nifedipine-resistant *I*_st_ component at 1 µM. Fitting the concentration–response relationship to the Hill equation gave a half-maximal inhibitory concentration (IC_50_) value of 118.5 ± 28.8 nM (n = 4, N = 4) in Ca_V_1.2^DHP−/−^ SAN cells, similar to the value of 129.1 ± 28.8 nM (n = 4, N = 4) in wild-type cells. Thus, it is unlikely that Ca_V_1.2 confers the structural basis for *I*_st_ sensitivity to DHPs. Of interest, the IC_50_ for *I*_st_ was close to that for *I*_Ca,L_ in Ca_V_1.2^DHP−/−^ SAN cells (104.2 ± 26.6 nM, n = 4, N = 4, Fig. 5A), suggesting that Ca_V_1.3 is responsible for the DHP sensitivity of *I*_st_.

## Discussion

Here we have demonstrated, for the first time, that voltage-gated L-type Ca_V_1.3 Ca^2+^ channels are essential for the expression of a DHP-sensitive, voltage-dependent Na^+^ conductance, previously described as *I*_st_. Our finding is based on the observations that (1) *I*_st_ is consistently identified in wild-type SAN cells but not in Ca_V_1.3-deficient cells; (2) block of *I*_st_ by nifedipine was unaffected upon ablation of Ca_V_1.2 DHP sensitivity in Ca_V_1.2^DHP−/−^ SAN cells; (3) DHP sensitivity of *I*_st_ overlapped that of Ca_v_1.3-mediated *I*_Ca,L_ in Ca_V_1.2^DHP−/−^ SAN cells; and (4) *I*_st_ could not be attributed to late *I*_Na_ or *I*_NCX_. Sensitivity to Ca^2+^-channel blockers such as DHPs, verapamil and diltiazem, as well as activators such as Bay-K8644 and FPL-64176, is based on highly specialized structural motifs conserved in Ca_V_1 α_1_-subunits of L-type Ca^2+^ channels^21, 31^. Thus, our genetic and pharmacological evidence showing overlapping properties between *I*_st_ and Ca_V_1.3-mediated *I*_Ca,L_ indicates a close functional relationship between these currents in SAN cells (Table 1). The demonstration of Ca_V_1.3 α_1_-subunits as essential molecular determinants of a voltage-dependent DHP-sensitive Na^+^ conductance is a novel and unexpected finding and constitutes a fundamental step in elucidating the molecular nature of *I*_st_.

Our patch-clamp recordings clearly show that under physiological conditions *I*_st_ is predominantly carried by Na^+^ rather than Ca^2+^ ions. Indeed, while lowering external Ca^2+^ did not affect *I*_st_, removal of extracellular Na^+^ abolished the current even in the presence of a physiological concentration of Ca^2+^ (Fig. 1). L-type Ca^2+^ channels are permeable to Na^+^ in the absence of extracellular divalent cations^32, 33^. However, L-type Ca^2+^ channels are highly selective for Ca^2+^ over Na^+^ with a permeation ratio (*P*_Ca_/*P*_Na_) of ~1000 under physiological conditions^32^. Therefore, Na^+^ influx through L-type Ca^2+^ channels is blocked by extracellular Ca^2+^ in the submicromolar range^32, 33^. It is thus unlikely that the “classical” permeation pathway of L-type Ca^2+^ channels mediates *I*_st_. Indeed, currently available recombinant Ca_V_1.3 channels with canonical channel pore sequence are Ca^2+^-selective *I*_Ca,L_, with poor permeability to Na^+^ at least in the experimental solutions used for the *I*_st_ recording in the present study (Toyoda *et al*., unpublished observation). Our results therefore suggest that Ca_V_1.3 α_1_-subunits in the SAN cell not only form Ca_V_1.3 L-type channels but also contribute to the formation of voltage-gated Na^+^ conductance through an unknown mechanism.

However, revealing the molecular mechanism allowing Ca_V_1.3 α_1_-subunits to form *I*_st_ is challenging. We favour the hypothesis that Ca_V_1.3 α_1_-subunits themselves form the *I*_st_ pore due to the observation that *I*_st_ and Ca_V_1.3 possess an essentially indistinguishable pharmacological profile and Ca^2+^-channel blockers have been shown to exert their pharmacological modulation exclusively by binding to α_1_-subunits^34^. In this case the different ion selectivity of *I*_st_ in SAN cells would require a modification of the ion permeation pathway. Substitution of negatively charged residues forming the ion selectivity filter of voltage-gated Ca^2+^ channels by lysine can indeed induce persistent Na^+^ currents similar to *I*_st_^35, 36^, suggesting that increased Na^+^ permeability *per se* could reproduce *I*_st_ properties. To date, analysis of Ca_V_1.3 transcripts has not identified alternatively spliced Ca_V_1.3 variants with a modified selectivity filter^37–39^. Since Na^+^ conductance through such modified channels may be larger than for Ca^2+^ ^36, 40^, *I*_st_ transcripts may be present at low levels. This would make their detection particularly difficult in tissues with low cell numbers such as the SAN. On the other hand, the possibility that *I*_st_ could be generated by alternative splicing of Ca^2+^ channels is also suggested by a recent report that T-type (Ca_V_3) Ca^2+^ channels of the snail heart have high permeability to Na^+^ due to unique splicing in the outer pore region^41^. Although this possibility cannot be excluded for mammalian SAN, splicing of T-type α_1_-subunits appears an unlikely explanation for *I*_st_ because of the L-type channel-specific pharmacology. Another possible explanation for Na^+^ selectivity of Ca_V_1.3 α_1_-subunits could be structural modifications of the ion conducting pathway through RNA editing, which so far has only been detected in the brain and in the cytoplasmic C-terminal tail of the channel^39, 42^.

Alternatively, a cationic channel functionally coupled to Ca_V_1.3 activity could mediate *I*_st_. In mouse SAN cells Ca_V_1.3 is co-localized with sarcoplasmic reticulum ryanodine receptors (RyRs) and controls diastolic RyR-dependent Ca^2+^ release^25, 43^. RyR-dependent Ca^2+^ release could then activate an inward Na^+^ current. However, the possibility that *I*_st_ is mediated by Ca^2+^-dependent opening of a cationic channel appears unlikely, because *I*_st_ density did not decrease upon lowering extracellular Ca^2+^ (Fig. 1), as one would expect for Ca^2+^-dependent opening of a Na^+^-selective channel associated with Ca_V_1.3. Finally, the possibility that *I*_st_ could be generated by direct opening of a Na^+^ channel physically coupled to Ca_V_1.3 channel gating is also unlikely, because *I*_st_ activates negative to Ca_V_1.3-mediated *I*_Ca,L_ (Fig. 1).

Ca_V_1.3 loss-of-function in mice or humans results in SAN dysfunction, which indicates that Ca_V_1.3 channels play a major role in pacemaker activity^11, 12, 44–46^. Consequently, the present findings also suggest that the loss of *I*_st_ could contribute to the SAN dysfunction induced by Ca_V_1.3 gene inactivation. In addition, our results indicate that the heart rate reducing effect of Ca^2+^ channel antagonists can be explained by drug binding to Ca_V_1.3 channels and reduction of Ca_V_1.3-mediated *I*_Ca,L_ and *I*_st_. Consistent with previous observations^11, 12^, our recordings in Ca_V_1.3^−/−^ SAN cells show that Ca_V_1.3 underlies a low-threshold *I*_Ca,L_ activated at voltages spanning the diastolic depolarization range. *I*_st_ differs from Ca_V_1.3-mediated *I*_Ca,L_ in the charge carrier and shows a more negative voltage for half-activation. *I*_st_ and Ca_V_1.3-mediated *I*_Ca,L_ could thus differentially contribute to the generation of the diastolic depolarization. For example, *I*_st_ could generate a persistent Na^+^ influx in the diastolic depolarization, while Ca_V_1.3-mediated *I*_Ca,L_ could generate inward Ca^2+^ current^12^ and control RyR-dependent Ca^2+^ release^43^. Notably, both *I*_st_ and Ca_V_1.3-mediated *I*_Ca,L_ are strongly potentiated by β-adrenergic activation, which suggests a dual role of Ca_V_1.3 in the sympathetic control of heart rate via *I*_Ca,L_ and *I*_st_.

In conclusion, we provide novel evidence supporting the involvement of Ca_V_1.3 in the generation of *I*_st_ in SAN cells. Our work provides valuable new insights into the molecular basis of *I*_st_ as well as the diverse functional significance of Ca_V_1.3 in cardiac pacemaker activity.

## Methods

### Ethics

The investigation conforms to the Guide for the Care and Use of Laboratory Animals (8^th^ edition, 2011), published by the US National Institutes of Health and European directives (2010/63/EU). The experimental protocol was approved by the Institutional Animal Care and Use Committee of Shiga University of Medical Science (Nos 2009-5-11, 2012-1-10 and 2014-12-3), the University of Montpellier and the University of Innsbruck.

### Ca_V_1.3^−/−^ and Ca_V_1.2^DHP−/−^ mice

Ca_V_1.3^−/−^ and Ca_V_1.2^DHP−/−^ mice were obtained by crossing mice from the original mutant colonies^28, 44^ with mice with a C57B6/J genetic background from Charles River in the animal facility, free of specific pathogenic organisms, of the Réseau d’Animalèrie de Montpellier (RAM) at the Institut de Génetique Humaine (Montpellier, France). We next backcrossed the offspring for 10 generations with C57B6/J mice before starting the study. Animals were given *ad libitum* access to food and drinking water and were maintained in a 12-h light–dark cycle (light, 8:30 a.m. to 8:30 p.m.). Only homozygous Ca_V_1.3^−/−^ and Ca_V_1.2^DHP−/−^ mice were used for the experiments.

### SAN cell preparations

Isolation of single SAN cells from mouse hearts was performed according to the methods of Mangoni and Nargeot^47^. Wild-type (N = 18), Ca_V_1.3^−/−^ (N = 10) and Ca_V_1.2^DHP−/−^ (N = 5) mice were anaesthetized with ketamine (100 mg/kg) combined with xylazine (10 mg/kg), and anticoagulated with heparin (250 units/mouse). Beating hearts were quickly removed and the SAN region was excised and cut into small strips in warm (35 °C) Tyrode solution containing (in mM): 140.0 NaCl, 5.4 KCl, 1.8 CaCl_2_, 1.0 MgCl_2_, 5.0 HEPES-NaOH and 5.5 D-glucose (adjusted to pH 7.4 with NaOH). The SAN tissue strips were then transferred to a low-Ca^2+^, low-Mg^2+^ solution containing (in mM): 140.0 NaCl, 5.4 KCl, 0.5 MgCl_2_, 0.2 CaCl_2_, 1.2 KH_2_PO_4_, 50.0 taurine, 5.5 D-glucose and 5.0 HEPES-NaOH with 1.0 mg/ml bovine serum albumin (BSA) (adjusted to pH 6.9 with NaOH), and then subjected to digestion by adding Liberase TH (0.1 mg/ml, Roche Diagnostics GmBH) and elastase (1.9 U/ml, Worthington Biochem. Co.) at 35 °C for a variable time of 9–14 min. Tissue strips were then transferred and washed in a Kraft-Bruhe (KB) solution containing (in mM): 70.0 L-glutamic acid, 20.0 KCl, 80.0 KOH, 10.0 (±) D-β-OH-butyric acid, 10.0 KH_2_PO_4_, 10.0 taurine and 10.0 HEPES-KOH, with 1 mg/ml BSA (pH adjusted to 7.4 with KOH). SAN cells were manually dissociated by agitation using a flame-forged Pasteur pipette in KB solution at 35 °C for ~5 min. Cellular automaticity was recovered by readapting the cells to physiological extracellular Na^+^ and Ca^2+^ concentrations by adding aliquots of solutions containing (in mM): 10.0 NaCl, 1.8 CaCl_2_ and, subsequently, normal Tyrode solution containing 1 mg/ml BSA. The final storage solution contained (in mM): 100.0 NaCl, 35.0 KCl, 1.3 CaCl_2_, 0.7 MgCl_2_, 14.0 L-glutamic acid, 2.0 (±) D-β-OH-butyric acid, 2.0 KH_2_PO_4_ and 2.0 taurine, with 1.0 mg/ml BSA (pH 7.4). Cells were harvested in custom-made recording Plexiglass chambers with glass bottoms for proper cell attachment and rinsed with normal Tyrode solution warmed to 36 °C just before patch-clamp recording.

### Whole-cell patch-clamp technique and data analysis

Isolated SAN cells were voltage-clamped using the whole-cell configuration of the patch-clamp technique with an EPC-8 patch-clamp amplifier equipped with an LIH-1600 AD/DA interface (HEKA) controlled by PatchMaster software or an Axon MultiClamp 700 A amplifier equipped with Digidata 1332 A interface-controlled PClamp software. Patch electrodes had a resistance of 2.5–4.0 MΩ when filled with the Cs^+^-rich intracellular solution containing (in mM): 125 CsOH, 20 tetraethylammonium chloride (TEA-Cl), 1.2 CaCl_2_, 5 Mg-ATP, 0.1 Li_2_-GTP, 5.0 EGTA and 10.0 HEPES (pH adjusted to 7.2 with aspartate). The concentration of free Ca^2+^ in the pipette solutions was calculated to be approximately 4.8×10^−8^ M (pCa = 7.3). The Cs^+^-substituted, K^+^-free external Tyrode solution contained (in mM): 140.0 NaCl, 5.4 CsCl, 1.8 CaCl_2_, 0.5 MgCl_2_, 0.33 NaH_2_PO_4_, 5.5 glucose and 5.0 HEPES (pH adjusted to 7.4 with NaOH). The concentration of CaCl_2_ in the external solution was reduced from 1.8 to 0.1 mM to separate *I*_st_ from *I*_Ca,L_. In some experiments, NaCl was totally substituted with NMDG-Cl, TEA-Cl or LiCl. All experiments were performed at 34–36 °C.

### Chemicals

Isradipine, nifedipine, verapamil, diltiazem, FPL-64176, Iso and ACh were purchased from Sigma-Aldrich. Drugs were prepared as 10 mM stock solutions in DMSO and then diluted in the external solution. TTX (Wako Chemical Co.) was dissolved in distilled water at a concentration of 10 mM and then diluted to the final concentration of 10 µM in the experimental solution.

### Statistical analysis

The results are expressed as mean ± S.E.M. Statistical comparison among the different groups was performed by one-way ANOVA followed by Tukey’s post-hoc HSD test. Statistical comparison between two groups was evaluated using Student’s t-test. N indicates the number of hearts and n indicates the number of cells used in experiments. A p value < 0.05 was considered statistically significant.

### Data availability

The datasets generated and/or analysed during the current study are available from the corresponding author upon request.
